# Oxidative DNA damage stalls the human mitochondrial replisome

**DOI:** 10.1038/srep28942

**Published:** 2016-07-01

**Authors:** Gorazd Stojkovič, Alena V. Makarova, Paulina H. Wanrooij, Josefin Forslund, Peter M. Burgers, Sjoerd Wanrooij

**Affiliations:** 1 Department of Medical Biochemistry and Biophysics, Umeå University, Umeå, Sweden; 2 Department of Biochemistry and Molecular Biophysics, Washington University School of Medicine, St. Louis, MO 63110, USA; 3 Institute of Molecular Genetics, Russian Academy of Sciences, Moscow 123182, Russia

## Abstract

Oxidative stress is capable of causing damage to various cellular constituents, including DNA. There is however limited knowledge on how oxidative stress influences mitochondrial DNA and its replication. Here, we have used purified mtDNA replication proteins, *i.e*. DNA polymerase γ holoenzyme, the mitochondrial single-stranded DNA binding protein mtSSB, the replicative helicase Twinkle and the proposed mitochondrial translesion synthesis polymerase PrimPol to study lesion bypass synthesis on oxidative damage-containing DNA templates. Our studies were carried out at dNTP levels representative of those prevailing either in cycling or in non-dividing cells. At dNTP concentrations that mimic those in cycling cells, the replication machinery showed substantial stalling at sites of damage, and these problems were further exacerbated at the lower dNTP concentrations present in resting cells. PrimPol, the translesion synthesis polymerase identified inside mammalian mitochondria, did not promote mtDNA replication fork bypass of the damage. This argues against a conventional role for PrimPol as a mitochondrial translesion synthesis DNA polymerase for oxidative DNA damage; however, we show that Twinkle, the mtDNA replicative helicase, is able to stimulate PrimPol DNA synthesis *in vitro*, suggestive of an as yet unidentified role of PrimPol in mtDNA metabolism.

The major function of mitochondria is to convert energy from nutrients into ATP through the process of oxidative phosphorylation (OXPHOS). Thirteen genes that encode for essential subunits of the OXPHOS system are located on the mitochondrial DNA (mtDNA), a small circular genome of 16 kb. Therefore it is not surprising that the accumulation of mtDNA molecules with mutations and/or deletions can lead to mitochondrial disease, characterized by decreased energy production^1^,^2^.

The process of energy production by the OXPHOS system inevitably generates some reactive oxygen species (ROS) that can cause oxidative damage to cellular components, including DNA. Frequent forms of ROS-induced DNA lesions include abasic sites (AP) and 8-oxo-7,8-dihydroguanine (8-oxo-G)^3^. There are several reasons why the effects of ROS on DNA might be especially deleterious in mitochondria. First, it has been proposed that, compared to nuclear DNA, mtDNA has a greater exposure to reactive oxygen species (ROS) due to its association with the mitochondrial inner membrane^4^,^5^ and thus its proximity to the OXPHOS system^6^,^7^,^8^. Second, in contrast to nuclear DNA, mtDNA replication is not restricted to S-phase and can therefore also take place when free nucleotide levels are relatively low^9^ and the redox environment of the cell is more oxidizing^10^ and thus more likely to cause oxidative damage to replicating DNA. These potential problems may be accentuated in quiescent (post-mitotic) cells that do not cycle through S-phase but still duplicate their mtDNA. Previously, the assumed lack of functional DNA repair mechanisms has been considered to contribute to the susceptibility of mtDNA to oxidative damage. However, multiple mtDNA repair pathways, including base excision repair that normally repairs both 8-oxo-G and abasic sites, have been described in the mitochondrial compartment^11^,^12^.

MtDNA is replicated by a unique machinery, parts of which are related to their counterparts in phage T7 13,14. The key factors include the heterotrimeric DNA polymerase γ (Pol γAB_2_) that consists of one Pol γA catalytic subunit and two processivity subunits (Pol γB), the mitochondrial single-stranded DNA binding protein (mtSSB), the DNA helicase Twinkle and the mitochondrial RNA polymerase (POLRMT) that functions as the primase in mtDNA replication^14^. Using these basic components, we have previously been able to reconstitute mtDNA replication *in vitro*^15^,^16^. During chromosomal DNA replication in the nucleus, both leading and lagging strand replicative polymerases (DNA polymerase ε and δ, respectively) stall when encountering oxidative DNA damage^17^. Specialized lesion bypass DNA polymerases are then recruited to synthesize past the oxidative DNA lesion and allow the replication fork to finish replication^18^. Surprisingly, given the wealth of information about nuclear DNA damage response mechanisms, very little is known about the fate of the mtDNA replication fork upon encountering DNA lesions caused by ROS. Human Pol γ has been reported to be unable to bypass abasic sites^19^, but was found to bypass 8-oxo-G in an error prone fashion^20^. Studies in the presence of the other components of the mtDNA replisome have to our knowledge not been carried out.

The recently discovered PrimPol enzyme has been proposed to participate in restarting replication during the nuclear DNA damage response^21^ and in both nuclear and mitochondrial translesion synthesis (TLS)^19^,^22^,^23^. Several groups have used recombinant PrimPol to characterize the properties of this enzyme^19^,^22^,^23^,^24^,^25^,^26^. These studies have shown that PrimPol’s primase activity can use both NTPs and dNTPs to synthesize primers *de novo*. Furthermore, PrimPol contains a polymerase activity that is able to efficiently bypass lesions such as 8-oxo-G and pyrimidine (6-4) pyrimidone photoproducts. However, the interaction of PrimPol with the components of the mitochondrial DNA replisome has not been studied, with the exception of a recent report that showed *in vivo* interaction between PrimPol and mtSSB^27^. In the present study we use a defined *in vitro* system to reveal the effects of specific oxidative DNA lesions on the mtDNA replication machinery. Our experiments show that an abasic site poses a complete block for mtDNA replication. Even 8-oxo-G causes pausing of mtDNA replication, in particular at lower dNTP concentrations. Surprisingly, we found PrimPol to be ineffective in mediating the bypass of AP or 8-oxo-G damage by the mtDNA replisome at “normal” dNTP concentrations. At “high” dNTP levels PrimPol could enhance replication by the mtDNA replisome, although this was DNA damage unspecific. Our results argue against a role for PrimPol as a TLS DNA polymerase for oxidative DNA damage in the mitochondria. However, we present that Twinkle, the mtDNA replicative helicase, is able to stimulate PrimPol DNA synthesis, thus suggesting PrimPol nonetheless plays an important role in mtDNA metabolism.

## Results

### Mitochondrial Pol γ stalls at oxidative DNA lesions

**Figure 1:**
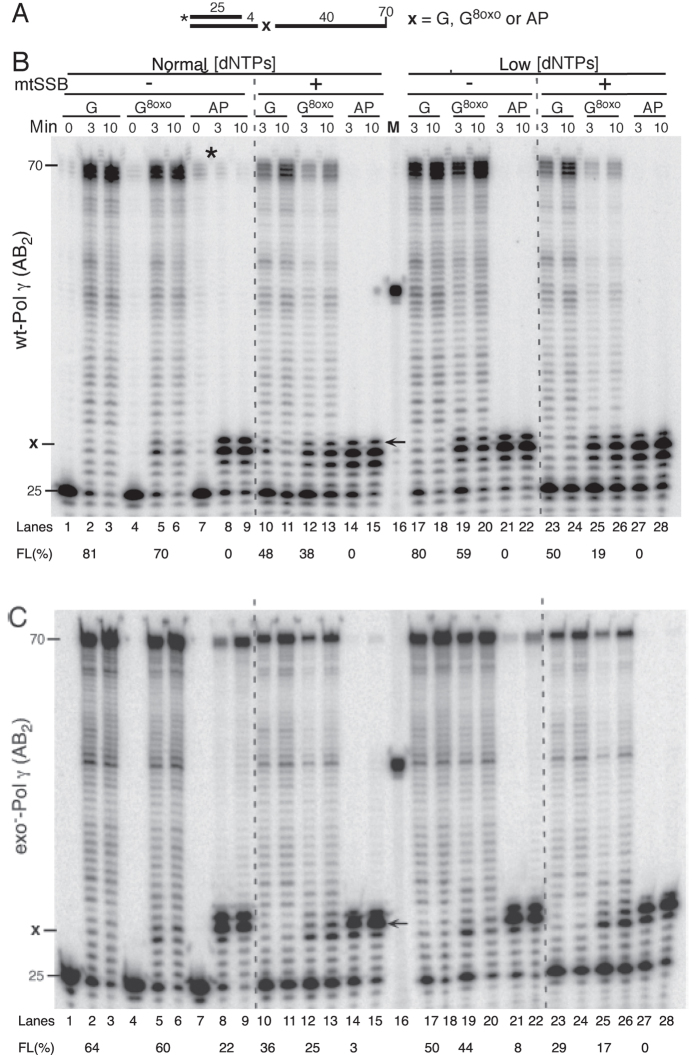
DNA pol γ translesion synthesis on oxidative DNA damage. (**A**) Schematic diagram of the oligonucleotide substrate. The template is a 70-mer. At the +5 position (indicated as ‘x’ and ‘←’) is either a dGTP (G), 8-oxo-G (G^8oxo^) or an abasic site (AP). The 70-mer DNA product represents full extension (full length = FL) of the 5′ ^32^P-labeled (*) 25-mer primer to the end of the template. The percentage of full-length product (% FL) is indicated below the 3 minute timepoints and was calculated by dividing the signal of the full-length products (68-70 nt) by the signal of the 25 nt input primer (lanes 1, 4 and 7 for non-damaged, 8-oxo-G and abasic site templates, respectively) on this specific gel. The experiment has been carried out three times; a representative experiment is shown. MtSSB (70 nM) was added where indicated. (**B**) Ten percent dPAGE of a time course reaction with exonuclease proficient Pol γA (12,5 nM) and Pol γB (18,75 nM) on undamaged (lane 1-3, 10-11, 17-18 and 23-24), 8-oxo-G (lane 4-6, 12-13, 19-20 and 25-26) or abasic site (lane 7-9, 14-15, 21-22 and 27-28) -containing DNA template. Reactions were performed in the presence of “normal” (lane 2-15) or “low” (lane 17-28) dNTP concentrations. The ‘*’ in lanes 7-8 indicates a carry over DNA product from lane 6. Note that this band is absent in lane 9, the longest time point. A repeat of the reactions in lanes 1-15 without this carryover is shown in Supplemental Fig. 1. (**C**) Gel image of a time course reaction with exonuclease-deficient Pol γA (12,5 nM) and Pol γB (18,75 nM) on undamaged (lane 1-3, 10-11, 17-18 and 23-24), 8-oxo-G (lane 4-6, 12-13, 19-20 and 25-26) or abasic site (lane 7-9, 14-15, 21-22 and 27-28) containing DNA template. Details are the same as in (**B**).

Oxidative stress is capable of causing damage to various cellular constituents, including DNA. To investigate how efficiently the mitochondrial DNA polymerase Pol γ bypasses oxidative DNA lesions, we performed *in vitro* DNA polymerization reactions with either wild type or the exonuclease-deficient D274A Pol γ holoenzyme (Pol γAB_2_) that lacks proofreading activity. The experiments were carried out on synthetic DNA templates containing either 8-oxoguanine (8-oxo-G) or an abasic site (AP). The first set of templates was designed such that Pol γ would encounter the DNA damage at the 5^th^ base after the initiation of DNA synthesis (Fig. 1A). The control substrates contained a template with an undamaged guanine in an otherwise identical sequence context.

Our aim was to perform the reactions at physiologically relevant dNTP concentrations. However, it is extremely difficult to determine the exact dNTP concentrations within cells and in particular inside the mitochondrial compartment^28^. Nevertheless, it is known that the different dNTPs are not equimolar and that dNTP concentrations are significantly lower in resting (non-dividing) cells than in cycling cells. Based on previously described values^28^,^29^, DNA replication reactions contained either 10 μM dTTP, 5 μM dCTP, 5 μM dATP and 3 μM dGTP to resemble levels in cycling cells (hereafter referred to as “normal” dNTP levels), or 2 μM dTTP, 1 μM dCTP, 2 μM dATP and 1 μM dGTP as an estimate of concentrations in resting cells (hereafter referred to as “low” dNTPs).

At “normal” dNTP concentrations, the wild type Pol γ holoenzyme was able to extend 81% of a 25-nucleotide end-labeled primer to a full-length product after a 3 min incubation with the undamaged DNA template (Fig. 1B, lane 2). Introduction of 8-oxo-G on the DNA template reduced the formation of full-length product to 70% and increased the replication pausing observed at nucleotide positions between +3 and +5 (Fig. 1B, compare lanes 2 and 5; see Supplemental Table 1 for quantification). Pol γ was completely blocked by an abasic site in the DNA template under these conditions (Fig. 1B, lanes 8-9). Because of the abundance of the mitochondrial single-stranded DNA-binding protein (mtSSB) in cells, single-stranded DNA replication intermediates will be coated with mtSSB^30^. Under our experimental conditions, the addition of mtSSB resulted in less full-length product (48% full-length, lane 10, compared to 81% in lane 2), but did not influence the relative efficiency of 8-oxo-G bypass (38% full length, lane 12). The presence of mtSSB had no effect on Pol γ translesion activity opposite an AP-site (lanes 14-15).

These reactions were performed at “normal” dNTP concentrations that aim to resemble the *in vivo* nucleotide pools in dividing/cycling cells^28^,^29^. However, unlike nuclear DNA, mitochondrial DNA is also duplicated in non-dividing tissues where the nucleotide pools are expected to be significantly lower^31^. Therefore, we performed identical reactions at about 5-fold lower dNTP concentrations (“low”) that are within the range expected to be found in quiescent cells^28^. While the lower dNTP concentrations did not appreciably affect DNA synthesis on an undamaged template (Fig. 1B, compare lane 17 to lane 2), pausing at the site of 8-oxo-G damage was modestly increased, both in the absence and in the presence of mtSSB (Fig. 1B, compare lane 5 with 19, and 12 with 25; Supplemental Table 1). As expected, no AP-site bypass by Pol γ was observed under “low” dNTP concentrations (Fig. 1B, lanes 21-22 and 27-28).

To investigate if proofreading by the catalytic Pol γ subunit influences bypass of oxidative damage, we performed identical experiments with an exonuclease-deficient (exo^−^) Pol γ mutant holoenzyme (Fig. 1C). Use of the exo^−^ Pol γ variant reduced the stalling at 8-oxo-G (compare Fig. 1B,C, lanes 5, 12, 19 and 25; Supplemental Table 1). Moreover, in contrast to the wild-type protein, exo^−^ Pol γ variant could carry out limited bypass of the abasic site (Fig. 1C, lanes 8-9, 14-15 and 21-22). However, as with wild type Pol γ, damage bypass by the exonuclease-deficient Pol γ was reduced at “low” dNTP levels and in the presence of mtSSB.

### The mtDNA replisome shows poor translesion synthesis past oxidative DNA damage

**Figure 2:**
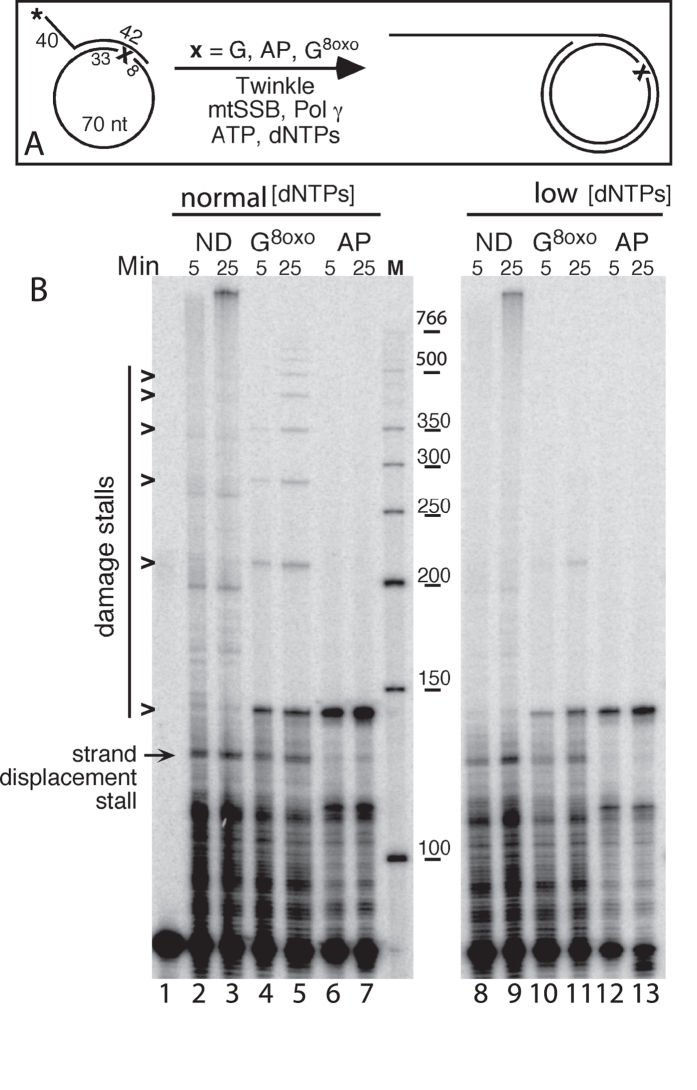
Oxidative DNA damage severely stalls the human mitochondrial replisome. (**A**) Scheme of the DNA replication assay. DNA polymerization was carried out with a 82 nts 5′ ^32^P (*) radiolabeled primer annealed to a 70-mer minicircle template. This generated a DNA substrate that has a 40 nts 5′ overhang to facilitate Twinkle loading and a 42 nts dsDNA region with at the -8 position (from the primer 3′ end) either a dGTP (G), 8-oxo-G (G^8oxo^) or an abasic site (AP) indicated as ‘x’. See Methods for details. (**B**) MtDNA replisome stalling at 8-oxo-G and abasic sites. The reactions were performed at 37 °C (5 and 25 min as indicated) in the presence of Twinkle (12.5 nM), mtSSB (250 nM), Pol γA (12.5 nM) and Pol γB (18.75 nM) and estimated physiological dNTP concentrations, “normal” (lane 2-7) or “low” (lane 17-28). The DNA template contains dGTP (ND lane 2-3 and 8-9), 8-oxo-G (lane 4-5 and 10-11) or an abasic site (lane 6-7 and 12-13). Stalling sites caused by the presence of oxidative DNA damage are indicated with arrowheads. The experiment has been carried out five times; a representative experiment is shown. A distribution plot of the signal in lanes 4 and 11 is presented in Supplemental Table 2A.

For processive replication of double-stranded mtDNA, Pol γ requires the activity of the DNA helicase Twinkle^32^. To examine the impact of Twinkle on the bypass of oxidative damage, we constructed a DNA substrate consisting of a primed minicircle with a 40 nt 5′ overhang to allow Twinkle loading (Fig. 2A). Once initiated, leading-strand DNA synthesis coupled to continuous unwinding of the dsDNA template can in theory continue indefinitely (rolling circle replication)^33^. On this template, the first encounter with oxidative DNA damage occurs after the extension of the primer to 144 nts. If the first lesion is bypassed, the mtDNA replisome encounters the same oxidative DNA damage every 70 nts. On a non-damaged template the mtDNA replisome was able to efficiently synthesize long fragments of ssDNA (longer than 766 bp, the highest band in the marker lane) and this replication was not substantially affected at “low” dNTP concentrations (Fig. 2B, compare lanes 2-3 with 8-9). When products longer than 766 nt were quantified relative to the input signal in lane 1, they represented 16% and 14% in lanes 3 and 9, respectively. At “normal” dNTP levels, 8-oxo-G stalled mtDNA replication, which resulted in the accumulation of DNA products with a 3′ end preceding the position of the lesion (at nt positions 144, 214, 274, 344 *etc*., lanes 4-5 in Fig. 2B). However, bypass of 8-oxo-G is evident and DNA fragments up to 500 nt in length were synthesized under these conditions (Fig. 2B, lanes 4 and 5). In contrast, at “low” dNTP concentrations, less bypass products (products over 144 nt) were observed on the 8-oxo-G containing template when compared to reactions carried out at “normal” dNTP concentrations and only short DNA products were observed even after prolonged incubation (Fig. 2B, lane 11; Supplemental Fig. 2). An abasic site presented a complete block for the mtDNA replisome even in the presence of Twinkle at both dNTP concentrations tested, as no bypass replication products were observed past the first abasic site (nt position 144, lanes 6-7 and 12-13 in Fig. 2B). To verify that this complete block of the mtDNA replisome was not an artifact due to a nick in the 70 nt minicircle, we repeated the experiment with the exo^−^ form of Pol γ, which is able to bypass an abasic site, albeit poorly (as shown in Fig. 1C, *e.g*. lane 22). On the minicircle DNA template, exo^−^ Pol γ could bypass the AP site, albeit inefficiently, resulting in the generation of DNA products representing up to five subsequent encounters of the abasic site in this rolling circle model (Supplemental Fig. 2).

### PrimPol does not significantly affect oxidative damage bypass by Pol γ

More oxidative damage is thought to occur on mtDNA than on nuclear DNA^34^. Our data therefore raises the question of how the mtDNA replisome would deal with oxidative damage if Pol γ is unable to bypass such a lesion, as shown above particularly in the case of abasic sites. A novel mammalian DNA polymerase, named PrimPol, has recently been described and reported to be localized to both the nucleus and the mitochondria^19^,^21^. PrimPol has been suggested to affect the bypass of oxidative DNA damage, including abasic sites^19^,^22^,^23^. Therefore, we purified PrimPol to test if it could assist the mitochondrial DNA replication machinery as a translesion synthesis (TLS) polymerase upon encounter of oxidative DNA damage.

**Figure 3:**
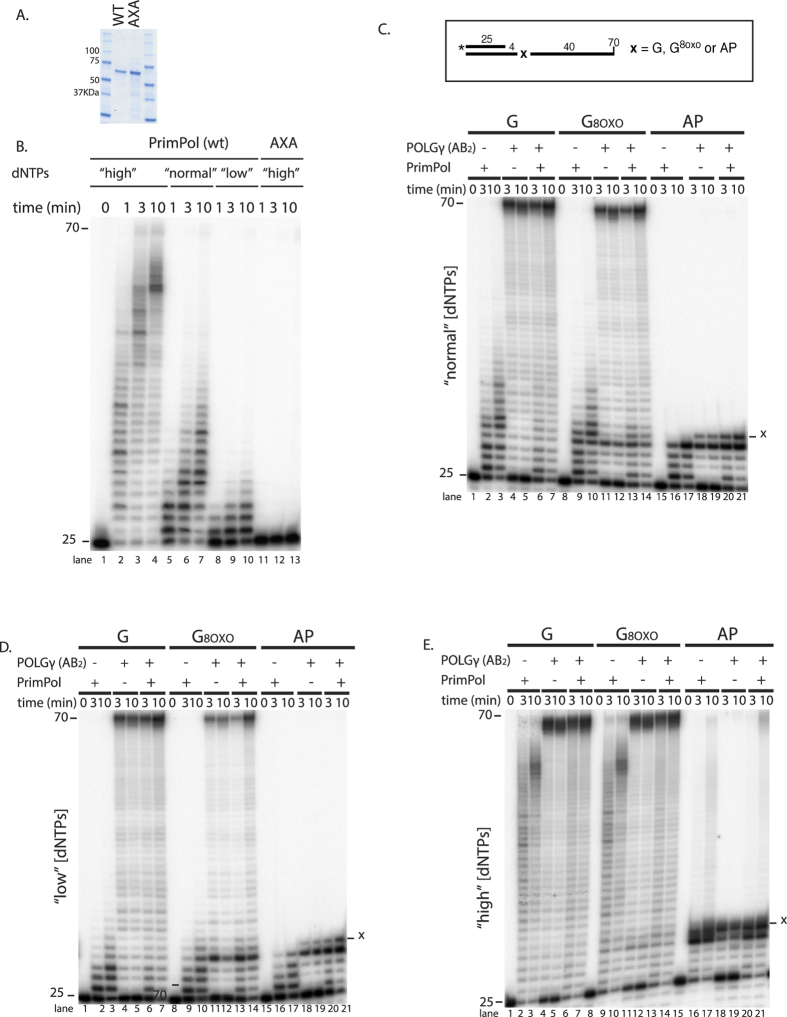
PrimPol’s translesion synthesis on oxidative DNA damage. (**A**) Coomassie Brilliant Blue stain of purified recombinant human PrimPol. Wild type (WT) or catalytically dead D114A/E116A mutant (AXA). (**B**) Ten percent dPAGE of a time course reaction with 200 nM wild type (WT) and catalytically dead PrimPol. DNA polymerization was carried out with a 25 nt 5′ ^32^P (*) radiolabeled primer annealed to a 70-mer linear template in the presence of “high” (lanes 2-4 and 11-13), “normal” (lanes 5-7) and “low” (8-10) dNTP concentrations. (**C–E**) Gel image of a time course replication reaction as indicated with Primpol (200 nM) or Pol γAB_2_ (12,5 nM) or both PrimPol (200 nM) and Pol γAB_2_ (12,5 nM) at (**C**) “normal”, (**D**) “low” and (**E**) “high” dNTP levels. The reactions were performed on undamaged (lanes 1-7, 8-oxo-G (lanes 8-14) or abasic site (lanes 15-21) containing DNA template. The experiments have been carried out three times; representative gels are shown. Quantification of (**C**–**E**) is presented in Supplemental Table 2; also see Supplemental Fig. 3.

Earlier studies on recombinant PrimPol were performed at a wide variety of experimental conditions^19^,^22^,^23^,^24^. We therefore optimized the purification strategy and reaction conditions for PrimPol (Boldinova *et al*., manuscript in preparation). For the purpose of this study, we chose to use Mg^2+^ as a divalent metal ion and omit Mn^2+^ in order to avoid confounding effects of the template-independent DNA synthesis activity that PrimPol has been reported to exhibit in the presence of Mn^2+^ 25. First, we analyzed the DNA polymerase activity of recombinant PrimPol at different dNTP concentrations (Fig. 3B). At “low” dNTP concentrations, PrimPol is able to extend the 25-nucleotide 5′ end-labeled primer on undamaged DNA with low processivity (Fig. 3B, lanes 8-10). Increasing the dNTP concentrations to “normal” levels enhanced the length of DNA fragments synthesized (Fig. 3B, compare lanes 8-10 to 5-7). Interestingly, further increase to “high” dNTP levels (equimolar 200 μM dNTPs) resulted in synthesis of longer DNA products that were able to reach the end of the template (Fig. 3B, lanes 2-4). Even though we estimate the purity of our recombinant PrimPol preparation to be >99% (Fig. 3A), we decided to test a catalytically dead (D114A/E116A) PrimPol mutant to exclude the possibility that the DNA synthesis observed was a result of a contaminant DNA polymerase activity from the *E. coli* strain used for expression. As expected, the D114A/E116A PrimPol, bearing mutations in the conserved active site metal binding residues, was unable to support DNA synthesis (Fig. 3B, lanes 11-13). These results support the conclusion that the observed DNA products are produced by PrimPol.

Next, we investigated how PrimPol replicates DNA that contains damaged bases. The analysis was carried out at three different dNTP concentrations: “normal” (Fig. 3C), “low” (Fig. 3D) and “high” (Fig. 3E). In agreement with previously published work^19^,^23^,^25^,^35^, 8-oxo-G did not impede DNA synthesis by PrimPol at “high” dNTP concentrations, as illustrated by the fact that the pattern of DNA products was comparable on non-damaged *vs*. 8-oxo-G template (Fig. 3E, compare lanes 2-3 with lanes 9-10; Supplemental Fig. 3). Furthermore, PrimPol’s ability to bypass this type of damage was also observed at “normal” and “low” dNTP concentrations (Fig. 3C,D, compare lanes 2-3 with lanes 9-10; Supplemental Fig. 3), something that has not been previously addressed.

If PrimPol were to function as a TLS polymerase also within the mitochondrial compartment, we hypothesized that it may be able to work together with Pol γ to improve bypass of DNA lesions. To test this idea, we carried out reactions containing either PrimPol or Pol γ alone, as well as their combination. Unexpectedly, the addition of PrimPol did not appreciably improve 8-oxo-G bypass by Pol γ (Fig. 3C–E, compare lanes 11-12 to 13-14; Supplemental Table 2). It should be noted that at the 10 min timepoint, a small increase in the amount of full-length (70 nt) DNA product was observed upon PrimPol addition; however, this increase was also seen on the undamaged template (Fig. 3C–E, *eg*. lanes 5 *vs*. 7 and 12 *vs*. 14; Supplemental Table 2). Moreover, primer usage was more efficient in the presence of PrimPol (*eg*. Fig. 3C, compare lane 12 *vs*. 14). Taken together, these observations are consistent with Pol γ extending products synthesized by PrimPol, In contrast, PrimPol appears to be unable to access the extended DNA primer upon Pol γ stalling, as evidenced by the fact that PrimPol did not reduce stalling of Pol γ at 8-oxo-G (Supplemental Table 2). Therefore, the data presented in Fig. 3 do not support a model where PrimPol would take over DNA synthesis upon stalling of Pol γ at 8-oxo-G damage.

Another possible role of PrimPol could be the reduction of the mutagenic potential of an 8-oxo-G bypass. To determine the base inserted by Pol γ or PrimPol opposite 8-oxo-G, we sequenced the longer DNA products (Fig. 3E lanes 10 and 12). Sequencing analysis showed that Pol γ incorporated the correct base, dC, opposite of 8-oxo-G in 73% of the sequenced products (not shown). The corresponding percentage for PrimPol was 53%, with all misincorporation events stemming from insertion of dA opposite the 8-oxo-G.

We also tested the ability of PrimPol to bypass an abasic site. Similarly to Pol γ, PrimPol was unable to bypass an abasic site at “normal” or “low” dNTP concentrations, both alone or in combination with Pol γ (Fig. 3C,D, lanes 16-17, 20-21). However, “high” dNTP levels stimulated PrimPol such that some bypass of the AP site was observed (Fig. 3E, lane 17). The longer DNA products in the reaction with both PrimPol and Pol γ suggest that Pol γ is able to extend the products that PrimPol synthesizes past the abasic site to produce full-length products (Fig. 3E, lane 21 compared to lane 17). However, as the AP-site bypass was only observed at the highest dNTP concentrations tested (200 μM), which are likely well beyond the range observed *in vivo*, the results of Fig. 3 are consistent with PrimPol providing very limited, if any, assistance to the mtDNA replicative polymerase in bypassing the two types of oxidative lesions tested.

### DNA Pol γB and mtSSB are unable to enhance PrimPol DNA synthesis on oxidative DNA damage

**Figure 4:**
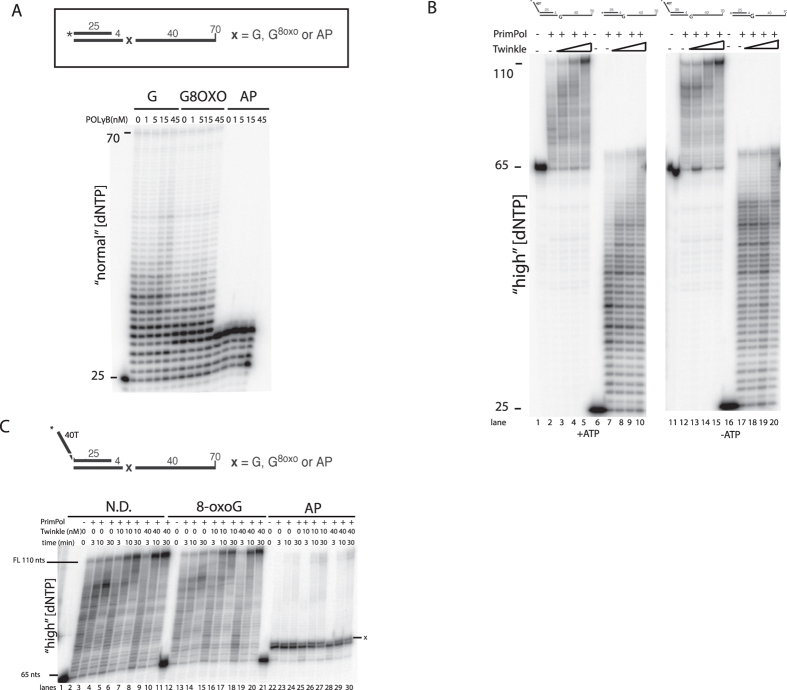
Twinkle stimulates PrimPol DNA synthesis *in vitro* in a DNA damage unspecific manner. (**A**) POL γB does not function as PrimPol processivity factor. DNA replication assay with 200 nM of PrimPol at “normal” dNTP concentrations with the addition of the indicated concentration of Pol γB dimer. The reaction were performed at 37 °C for 20 min. (**B**) At “high” dNTP concentrations Twinkle leads to an increase in the length of DNA fragments synthesized by PrimPol. The DNA template used consisted of a primed 70 nt linear template with a 40 nt 5′ overhang to allow Twinkle loading and a dsDNA region of 25 nts. PrimPol (200 nM) DNA synthesis assay with 0, 5, 10 or 40 nM of Twinkle on a template without and with a 40 nt poly-dT (40T) 5′ overhang to facilitate Twinkle loading. Reactions where performed with or without ATP (4 mM). (**C**) Twinkle stimulates DNA synthesis by PrimPol in a DNA damage unspecific fashion. Primer extension of 200 nM PrimPol on undamaged (**G**), 8-oxo-G or abasic site (AP) containing DNA templates at “high” dNTP concentrations. Reactions were run in the presence indicated amount of Twinkle and stopped at the indicated time points as described in the Methods. These experiments have been carried out 2–3 times; a representative experiment is shown.

As PrimPol did not appear to assist Pol γ in bypass, we next decided to explore possible functional interactions of PrimPol with other components of the mtDNA replisome. During standard mtDNA replication, Pol γB acts as a processivity factor, accelerating the polymerization rate and enhancing the affinity of the Pol γA catalytic subunit for DNA^36^. To investigate whether it could play a similar role for PrimPol, we performed primer extension experiments with PrimPol in the presence of increasing amounts of Pol γB. As shown in Fig. 4A, Pol γB did not affect the DNA synthesis by PrimPol on an undamaged template nor on a template with 8-oxo-G or an abasic site. POL γB does therefore not function as a processivity factor for PrimPol.

PrimPol has been reported to interact with mitochondrial single-stranded DNA binding protein (mtSSB)^27^. In agreement with earlier reports^27^,^35^, the addition of 25 nM mtSSB inhibited DNA synthesis by PrimPol, giving rise to shorter products (Supplemental Fig. 4). The average length of the DNA products synthesized by PrimPol decreased further when mtSSB was increased to 125 nM (full coating of the available ssDNA is estimated at 5 nM of tetrameric mtSSB). Mitochondrial SSB did not differentially influence DNA synthesis by PrimPol on a damage-containing DNA template (Supplemental Fig. 4).

### Twinkle alters PrimPol’s DNA synthesis in a DNA damage unspecific manner

If PrimPol participates in mtDNA metabolism, it is likely to interact with Twinkle, the replicative DNA helicase and a constitutive part of the mitochondrial nucleoids^32^. To test whether Twinkle influences DNA synthesis by PrimPol, we prepared a DNA template consisting of a primed 70 nt linear template with a 40 nt 5′ overhang that allows Twinkle loading (Fig. 4B). The substrate is not unwound, since it lacks a ssDNA 3′-tail required for Twinkle helicase activity^37^. The 5′ overhang did not affect PrimPols ability to perform primer extension (compare Fig. 4B, lane 2, with *e.g*. Fig. 3B). To our surprise, at “high” dNTP levels Twinkle stimulated the synthesis of longer DNA fragments by PrimPol (Fig. 4B). The influence of Twinkle was dose dependent (Fig. 4B, lanes 2-5) and independent of ATP (Fig. 4B, lanes 12-15). The stimulation required loading of Twinkle onto the substrate, because it was less pronounced on a DNA substrate without a 5′ overhang (Fig. 4B, lanes 7-10 and 17-20). Twinkle showed similar stimulation of DNA synthesis by PrimPol on both undamaged and damaged templates, as judged by the amount of full-length DNA product (Fig. 4C). The Twinkle dependent stimulation for PrimPol DNA synthesis was not observed at “normal” dNTP concentrations; nonetheless, the results at “high” dNTP concentrations indicate a functional interaction between these two proteins.

### PrimPol does not enhance bypass of oxidative DNA damage by the mtDNA replisome

**Figure 5:**
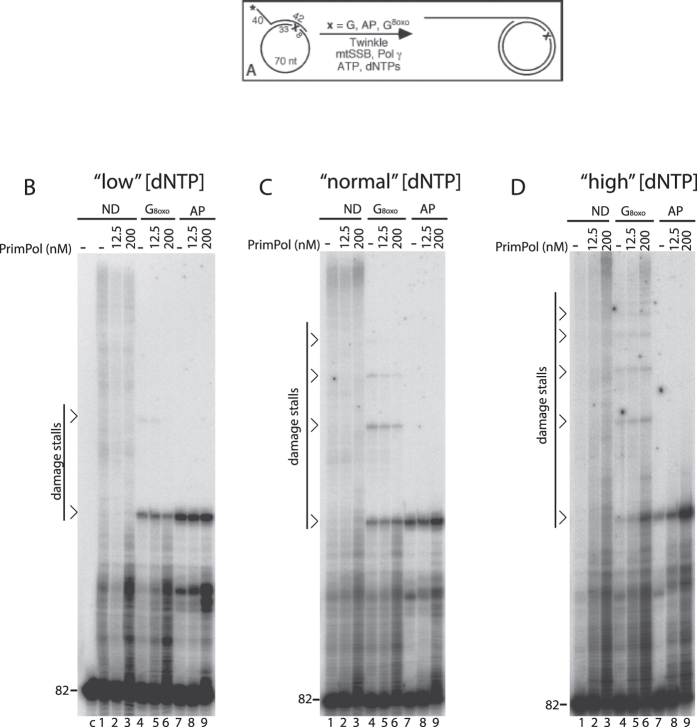
PrimPol does not function as lesion bypass polymerase at the mtDNA replication fork. (**A**) Scheme of the DNA replication assay. DNA polymerization was carried out with a 82 nt 5′ ^32^P (*) radiolabeled primer annealed to a 70-mer minicircle template. This generated a DNA substrate that has a 40 nts 5′ overhang to facilitate Twinkle loading and a 42 nts dsDNA region with at the -8 position (from the primer 3′ end) either a dGTP (**G**), 8-oxo-G (G^8oxo^) or an abasic site (AP) indicated as ‘x’. (**B**–**D**). Rolling circle replication reactions in the presence of PrimPol. The reactions were performed at 37 °C for 25 min in the presence of Twinkle (12.5 nM), mtSSB (25 nM), Pol γA (12.5 nM) and Pol γB (18.75 nM) at 3 different dNTP concentrations, “low” (**B**), “normal” (**C**), or “high” (**D**) and with the indicated amount of PrimPol. The DNA template contained dG (ND), 8-oxo-G (G^8oxo^) or an abasic site. Stalling sites caused by the presence of oxidative DNA damage and the migration of the 62 nt primer are indicated. Lane “c” in (**B**): input DNA template without addition of any protein. The experiment has been carried out three times; a representative experiment is shown. Quantification of bypass products is presented in Supplemental Table 3.

Finally, given the above effect of Twinkle on PrimPol, we investigated whether PrimPol could facilitate the bypass of oxidative damage in co-operation with the mitochondrial replisome (Pol γAB_2_, mtSSB and Twinkle). In this model, we hypothesized that PrimPol might assist in bypass either by inserting across the DNA damage or by extending the mismatched primer after insertion across damage by Pol γ. We used the same DNA substrate with a 40 nt 5′ overhang as in Fig. 2; on this template, the first encounter with oxidative DNA damage occurs after the extension of the primer to 144 nts (Fig. 5A). The reactions with the mitochondrial replisome were performed with increasing amounts of PrimPol.

As was found in Fig. 1 for Pol γ alone, the mtDNA replisome showed substantial replication stalling at 8-oxo-G and abasic sites (Fig. 5B, lanes 4 and 7). For each lane, we quantified the percentage of signal intensity derived from DNA products that had been extended past the first damage encounter (hereafter referred to as “bypass products”). On the non-damaged template, bypass products comprised 22% of total signal; this decreased to 2% and 0% on the 8-oxo-G and abasic site templates, respectively (quantified from Fig. 5B, lanes 1, 4 and 7; see Supplemental Table 3 for quantification). As expected, more 8-oxo-G bypass products were observed at higher dNTP concentrations (lane 4 in Fig. 5B–D; 2%, 7% and 13% bypass products at “low”, “normal” and “high” dNTPs, respectively). At “low” and “normal” dNTP concentrations, the addition of PrimPol to the replisome did not have a considerable effect on the level of bypass, and only lead to increased accumulation of short DNA products (Fig. 5B,C, compare lanes 6 and 9 to lane 3). At optimal conditions for PrimPol (“high” dNTPs), addition of PrimPol increased the overall amount of DNA products from all templates but did not specifically improve damage bypass (Fig. 5D, lanes 4-6 and 7-9; Supplemental Table 3). No bypass of the abasic site was observed at the dNTP concentrations tested (Fig. 5B–D, lanes 7-9). In conclusion, although addition of PrimPol gave rise to more replication intermediates at “high” dNTP levels, our data does not support a conventional role of PrimPol as a mitochondrial translesion polymerase at sites of oxidative damage.

## Discussion

The integrity of the mtDNA is essential for proper cellular energy metabolism. Reactive electrophilic oxygen species are byproducts of mitochondrial respiration and can damage mtDNA, contributing to mutagenesis and mitochondrial disease^38^. Despite the presence of some repair mechanisms that target and repair oxidatively damaged DNA bases^39^,^40^, it is unavoidable that some oxidative damage will persist long enough to be encountered by the mtDNA replication machinery. For example, between 1 and 7000 8-oxo-Gs are thought to persist for every million guanines in mammalian mtDNA^34^,^41^. Therefore, it is of great interest to understand how the mitochondrial replisome deals with oxidative damage.

In this study, we used DNA templates comprising of synthetic oligonucleotides to determine the action of the various mtDNA replication proteins upon encounter of oxidative DNA damage (8-oxo-G or abasic sites). At “normal” dNTP concentrations (similar to those present in cycling cells), DNA polymerase γ shows moderate stalling at 8-oxo-G sites, in particular when the DNA is coated with mtSSB as is expected to be the case *in vivo* (Fig. 1B). The same is true for the mitochondrial replisome that includes the Twinkle helicase (Fig. 2). The regulation of cellular dNTP levels is synced with the needs of nuclear DNA replication so that dNTP levels are high in S-phase but significantly lower outside of S-phase and in non-cycling cells. However, mitochondrial DNA undergoes duplication even in non-dividing cells, and is thus forced to replicate at the lower dNTP concentrations that prevail. For this reason, we also carried out our analysis at “low” dNTP concentrations meant to mimic those found in resting cells^42^. We found the stalling of the mtDNA replisome at 8-oxo-G to be further exacerbated under these conditions (Fig. 2B, right panel). This observation suggests that oxidative stress can lead to mtDNA instability, particularly in non-dividing cells.

The replication pausing at 8-oxo-G suggests that Pol γ detects the 8-oxo-G damaged nucleotide, which has potential ramifications for the mitochondrial mutation spectrum because 8-oxo-G can mispair with A resulting in G > T and C > A transversions. Pol γ’s ability to distinguish 8-oxo-G could, for example, explain the lack of a G > T bias in mtDNA in aged humans and mice^43^,^44^ or in *Drosophila melanogaster* with elevated levels of 8-oxo-G^45^. Because Pol γ is inefficient at bypassing 8-oxo-G, some other mechanism must exist for coping with this type of DNA lesion in the mitochondria. Given our observation that PrimPol is more error-prone than Pol γ when inserting across 8-oxo-G (inserting an A instead of a C 47% of the time), PrimPol is not a likely candidate. Moreover, although our data confirm previous reports regarding the robust bypass of 8-oxo-G by PrimPol^19^,^23^,^25^,^35^, this polymerase was not able to assist Pol γ stalled at 8-oxo-G (Fig. 3C–E), even in the presence of Twinkle (Fig. 5B–D).

An abasic site was found to pose an absolute block for the proofreading proficient mtDNA replisome (Figs 1B and 2). Likewise, PrimPol alone or in combination with the mtDNA replisome was unable to bypass this type of damage (Figs 3C,D and 5B,C) at “normal” dNTP concentrations. Even at “high” dNTP concentrations, bypass of the AP-site was very inefficient (Figs 3E and 5D; 2% bypass products in lane 9 of 5D). These findings are in line with a number of previous reports showing little or no bypass of AP-sites by PrimPol^19^,^23^,^25^. In contrast, others have reported PrimPol-dependent bypass of AP-sites when manganese is used as the cofactor^19^,^26^. The “high” dNTP concentrations used in this study correspond to 200 μM of each dNTP. Such high dNTP pools are unlikely to be found within the cell, as even when dNTP synthesis is boosted in response to DNA damage the increase is limited to 5- or 2-fold over normal levels in *S. cerevisiae* or mammalian cells, respectively^46^,^47^.

Taken together, our results argue against a conventional role for PrimPol as a mitochondrial lesion bypass DNA polymerase for oxidative DNA damage. However, we cannot exclude the possibility that factors such as posttranslational modifications or interaction with accessory proteins could modulate the efficiency of bypass of oxidative damage by PrimPol within mitochondria. Although no PrimPol accessory subunits have been identified, other lesion bypass polymerases are significantly more efficient when forming multisubunit complexes (*e.g.*, Pol ζ 48). Neither does our data exclude a role for PrimPol in oxidative mtDNA damage tolerance through other mechanisms such as template switching^49^ or repriming of mtDNA replication to allow replication restart^26^. In future studies, we will attempt to address whether PrimPol functions through these yet speculative mechanisms in the mitochondria.

It is interesting that addition of Twinkle to the PrimPol reaction at “high” dNTP levels leads to longer DNA replication products (Fig. 4B). The observed effect requires the loading of Twinkle on the DNA substrate as shown by a more modest stimulation on a template lacking the 5′ overhang that facilitates Twinkle loading (Fig. 4B). Furthermore, preincubation of Twinkle with the DNA template accelerates the stimulatory effect on PrimPol (data not shown). Finally, oxidative DNA damage bypass synthesis was most efficient in the presence of Twinkle, although the stimulation by Twinkle was not specific to damaged DNA and was also observed on undamaged template (Fig. 4C). At this stage, we cannot explain the effect of Twinkle on PrimPol activity but the functional interaction between these two proteins is intriguing and well worth more detailed study.

## Methods

### Purification of recombinant proteins

TWINKLE, mtSSB, PolγA and PolγB were expressed and purified as previously described (13,28). For the preparation of exonuclease-deficient PolγA, a D274A mutation was introduced into a 6xHIS-tagged version of POLγA in a pBacPAK9 plasmid by PCR-based mutagenesis and used to prepare an *Autographa california* nuclear polyhedrosis virus recombinant for the proteins as described in the BacPAK manual (Clontech). The exonuclease-deficient PolγA variant was purified following the same protocol used for the wild-type PolγA (14). The full-length cDNA of the CDCC111 human gene was obtained from Origene (http://www.origene.com).

A DNA fragment encoding for the PrimPol gene was PCR-amplified and cloned into the pGEX-6-P1 plasmid in frame with the N-terminal GST-tag using the BamHI and SalI restriction sites. *E. coli* ArcticExpress (DE3) cells were transformed with the expression plasmid PrimPol-pGEX-6-P1. An overnight culture was grown at 30 °C and used to inoculate 2 liters of LB-medium. The culture was grown at 30 °C to an OD_600_ = 0.8, then the temperature was reduced to 12 °C and expression induced with 0.5 mM IPTG for 18 hours.

Cells were harvested by centrifugation at 4,000 × g for 15 min at 4 °C. Pellets were first washed and then resuspended in lysis buffer A1 (50 mM HEPES pH 7.4, 8% glycerol, 500 mM NaCl, 1 mM DTT, 0.1% Tween 20, 0.01% Brij 58, 1 μM Pepstatin, 0.1 mM AEBSF, 1 mM DTT and 1 mM E64) and pulse-sonicated with 15 × 10 s. Cell lysates were clarified by centrifugation at 25.000 g for 30 min at 4 °C and incubated with glutathione sepharose beads (GE Healthcare) overnight at 4 °C. The beads were washed with buffer A1, followed by washing with buffer A2 (50 mM HEPES pH 7.8, 8% glycerol, 500 mM NaCl, 1 mM DTT, 0.1% Tween 20, 0.01% Brij 58, 1 μM Pepstatin, 0.1 mM AEBSF, 1 mM DTT and 1 mM E64). A second wash with buffer A2 without protease inhibitors was performed, followed by elution of the protein with buffer A3 (50 mM Hepes pH 7.8, 8% glycerol, 500 mM NaCl, 0.1% Tween 20, 0.01% Brij 58, 1mM DTT and 30 mM reduced glutathione). Fractions containing PrimPol were combined and digested overnight at 4 °C with 50 μL of 1 mg/mL PreScission protease to remove the GST tag. The eluate was dialyzed against buffer B1 and loaded onto a Heparin HP 1 mL column (GE healthcare). After washing the column with buffer B1 (50 mM Hepes pH 7.4, 2% glycerol, 1 mM DTT, 180 mM NaCl, 0.0025% Brij 58), a linear salt gradient from buffer B1 to buffer B3 (30 mM Hepes pH 7.4, 2% glycerol, 1 mM DTT, 500 mM NaCl) was used to elute the protein. The protein was concentrated using a Vivaspin 2 column and further purified on a Superdex 200 Increase 10/300 GL gel filtration column with B1 buffer used as a mobile phase. Protein samples were frozen in liquid N_2_ and stored at −80 °C.

### Preparation of DNA substrates

The 70-mer oligonucleotides with either a deoxyguanosine triphosphate (non-damaged), 8-oxo-G or AP-site (70 ND, 70 ^8oxo^G and 70 AP, respectively) as nt 30 from the 3′end (underlined) were ordered from Oligos Etc. and used as templates in primer extension assays. For rolling circle assays, the same linear oligonucleotide was converted to a single-stranded circular molecule by first phosphorylating and then hybridizing it to a 28-mer oligonucleotide, which was complementary to 14 bases at both ends of the 70-mer and thus promoted the covalent circularization of the 70-mer by T4 DNA ligase. The ligase reaction was stopped with 25 mM EDTA, the reaction mixture extracted with phenol/chloroform and the oligonucleotides recovered by ethanol precipitation. Approximately 75% of the reaction products were monomeric circles and they were purified by electrophoresis (10% polyacrylamide gel with 8 M Urea in TBE). Three different oligonucleotides to be used as primers for primer extension assays were purchased from Integrated DNA Technologies: a 25-mer, a 68-mer and a 82-mer. The primers were labeled at their 5′ end with γ[^32^P]ATP. In Supplemental Fig. 4A, 5′TET labeled 25-mer with identical sequence was used. For the primer extension assays, the ^32^P labeled 25-mer or 68-mer primers were annealed to the 70-mer linear templates. For the rolling circle replication assays the ^32^P labeled 68-mer and 82-mer primers were annealed to the single-stranded circular templates.

### Primer extension reaction with Pol γ

Reaction mixtures (10 μL) contained 25 mM Tris-HCl pH 7.5, 1 mM DTT, 10 mM MgCl_2_, 100 μg/ml BSA, 18.75 nM PolγB and 5 nM radiolabeled DNA substrate. Additionally we added either wild type (wt) (Fig. 1B) or exonuclease deficient (Fig. 1C) 12.5 nM PolγA to the reaction. As indicated, we added 70 nM of mtSSB. Full coating of the ssDNA is expected at 5 nM mtSSB^50^. The reactions were started by the addition of dNTPs. The concentrations used were 10 μM dTTP, 5 μM dCTP, 5 μM dATP and 3 μM dGTP (“normal”) or 2 μM dTTP, 1 μM dCTP, 2 μM dATP and 1 μM dGTP (“low”). After 3 and 10 min incubations at 37 °C, the reactions were stopped by the addition of 1.1 μL of a termination mixture (5% SDS, 250 mM EDTA) and analyzed on a 10% polyacrylamide 8 M urea gel. Quantification was performed by phosphorimaging of the dried gel (^32^P) on a Typhoon system. For each reaction, the percentage of primer that was extended to full-length product was calculated as the ratio between the signal of the full-length products (68-70 nts in length) and the signal of the 25-nt primer in the control reaction without protein (Fig. 1B,C; lanes 1, 4 and 7 for non-damaged template, 8-oxo-G and abasic site templates, respectively). Similarly, when signal at the pausing site was quantified (Supplemental Table 1), the signal density at nt positions +3, +4 and +5 was divided by the signal of the 25-nt primer in the control reaction. When Pol γ primer extension activity was tested alongside PrimPol (Fig. 3C–E), identical conditions as described for DNA synthesis reactions with PrimPol. 12.5 nM PolγA and 18.75 nM PolγB were used in those reactions.

### *In vitro* DNA synthesis with the mtDNA replisome

The 5′ end labeled primer on a mini-circle template (5 nM) was added to a reaction mixture (10 μL) containing 25 mM Tris-HCl (pH 7.5), 75 mM NaCl, 10 mM magnesium acetate, 1 mM DTT, 100 μg/ml BSA, 4 mM ATP, PolγA (12.5 nM), PolγB (18.75 nM, calculated as dimer), mtSSB (250 nM) and Twinkle (12.5 nM). The reactions were started by the addition of dNTPs with the indicated concentrations. When *in vitro* replisome DNA synthesis was performed with the addition of PrimPol (Fig. 5B–D), the reaction tube containing 10 mM Bis-Tris-Propane-HCl (pH 7.0), 10 mM MgCl_2_ and 1 mM DTT. 5 nM radiolabeled DNA substrate was added to the reaction mixtures (10 μL total volume). The reactions were performed at 37 °C and started by addition of proteins. At the indicated time points, the reactions were stopped by the addition of 1.1 μL of termination mixture (5% SDS, 250 mM EDTA) and analyzed on a 8% polyacrylamide gel containing 8 M urea. Quantification was performed by phosphorimaging of the dried gel (^32^P) on a Typhoon 9400 system (GE Healthcare). For Fig. 5, we quantified the percentage of bypass products by dividing the signal of DNA products larger than 150 nt by the total signal of the entire lane.

### DNA synthesis reactions with PrimPol

Primer extension reactions with linear or circular templates were performed in a buffer containing 10 mM Bis-Tris-Propane-HCl (pH 7.0), 10 mM MgCl_2_ and 1 mM DTT. 5 nM radiolabeled DNA substrate was added to the reaction mixtures (10 μL total volume) as well as dNTPs at 3 different concentrations: “low” (2 μM dTTP, 1 μM dCTP, 2 μM dATP and 1 μM dGTP), “normal” (10 μM dTTP, 5 μM dCTP, 5 μM dATP and 3 μM dGTP) or “high” (200 μM equimolar). The reactions were performed at 37 °C and started by addition of 200 nM PrimPol. At the indicated time points, the reactions were stopped by the addition of 1.1 μL of termination mixture (5% SDS, 250 mM EDTA) and analyzed on a 8–12% polyacrylamide gel containing 8 M urea. Quantification was performed by phosphorimaging of the dried gel (^32^P) on a Typhoon 9400 system (GE Healthcare).

### Fidelity analysis on 8-oxo-G containing template

The protocol was adapted as described previous^51^. Briefly, annealing of a biotinylated primer with internal HindIII site (BioHindIII, Supplemental Table 4) and a 70 nts template containing dGTP or 8-oxo-G (70 ND and 70 ^8oxo^G) was performed at 1:1 molar ratio. Primer extension reactions were performed as described for PrimPol DNA synthesis with the alterations that reactions were stopped by 1 h incubation at 70 °C and the products were immobilized for 15 min in room temperature on Dynabeads^TM^ M-280 Streptavidin following the manufacturer’s instructions. Separation of the newly synthesized strand from the template strand was performed by 2 consecutive incubations in 0.1 M NaOH (5 min), followed by washing (manufacturer’s instructions). The single stranded product was amplified by PCR using Phusion^®^ High-fidelity DNA polymerase (New England Biolabs), and primers (Supplemental Table 4). After purification, the 104 bp PCR product was cleaved (HindIII, BfaI) and ligated in pUC19 and transformed into *E. coli*. Colonies were picked and sequenced using the M13 (-49) primer (Supplemental Table 4).

## Additional Information

**How to cite this article**: Stojkovič, G. *et al*. Oxidative DNA damage stalls the human mitochondrial replisome. *Sci. Rep*. **6**, 28942; doi: 10.1038/srep28942 (2016).

## Supplementary Material

Supplementary Information
