# Supplementary material for: Oxidative DNA damage stalls the human mitochondrial replisome
Source: Sci Rep. 2016 Jul 1;6:28942. doi: 10.1038/srep28942 (PMC4929447; doi:10.1038/srep28942)
Supplement: Supplementary Information [file srep28942-s1.pdf]

Supplemental information

Oxidative DNA damage stalls the human mitochondrial replisome

Gorazd Stojkovič[1,4], Alena V. Makarova[2,3,4], Paulina H. Wanrooij[1,2], Josefin Forslund[1], Peter M. Burgers[2] and Sjoerd Wanrooij[1,2*]

1. Department of Medical Biochemistry and Biophysics, Umeå University, Umeå, Sweden.
2. Department of Biochemistry and Molecular Biophysics, Washington University School of Medicine, St. Louis, MO 63110, USA.
3. Institute of Molecular Genetics, Russian Academy of Sciences, Moscow 123182, Russia.
4. These authors contributed equally to this work.

* Correspondence Sjoerd.Wanrooij@umu.se

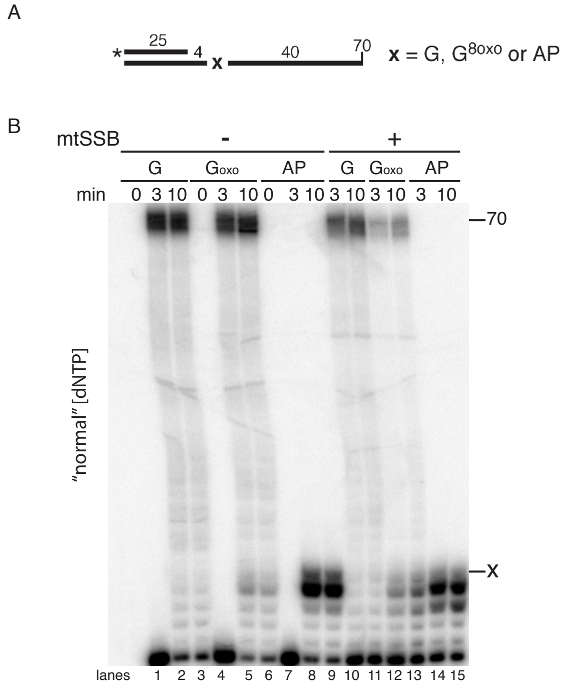

Supplemental Figure 1. Accompanies figure 1. DNA pol γ translesion synthesis on oxidative DNA damage at "normal" dNTP concentrations. A) Schematic diagram of the oligonucleotide substrate. The template is a 70-mer. At the +5 position (indicated as 'x') is either a dGTP (G), 8-oxo-G (G^{8oxo}) or an abasic site (AP). MtSSB (70 nM) was added where indicated. B) Ten percent dPAGE of a time course reaction with exonuclease proficient Pol γA (12,5 nM) and Pol γB (18,75 nM) on undamaged (lane 1-3, 10-11), 8-oxo-G (lane 4-6, 12-13) or abasic site (lane 7-9, 14-15) -containing DNA template. This experiment is identical to the reactions shown in Figure 1B (lanes 1-15), but is included in order to demonstrate that the DNA products marked with an asterisk "*" in Figure 1B (lanes 7-8) are the result of carry-over from lane 6, as they are not present in this image.

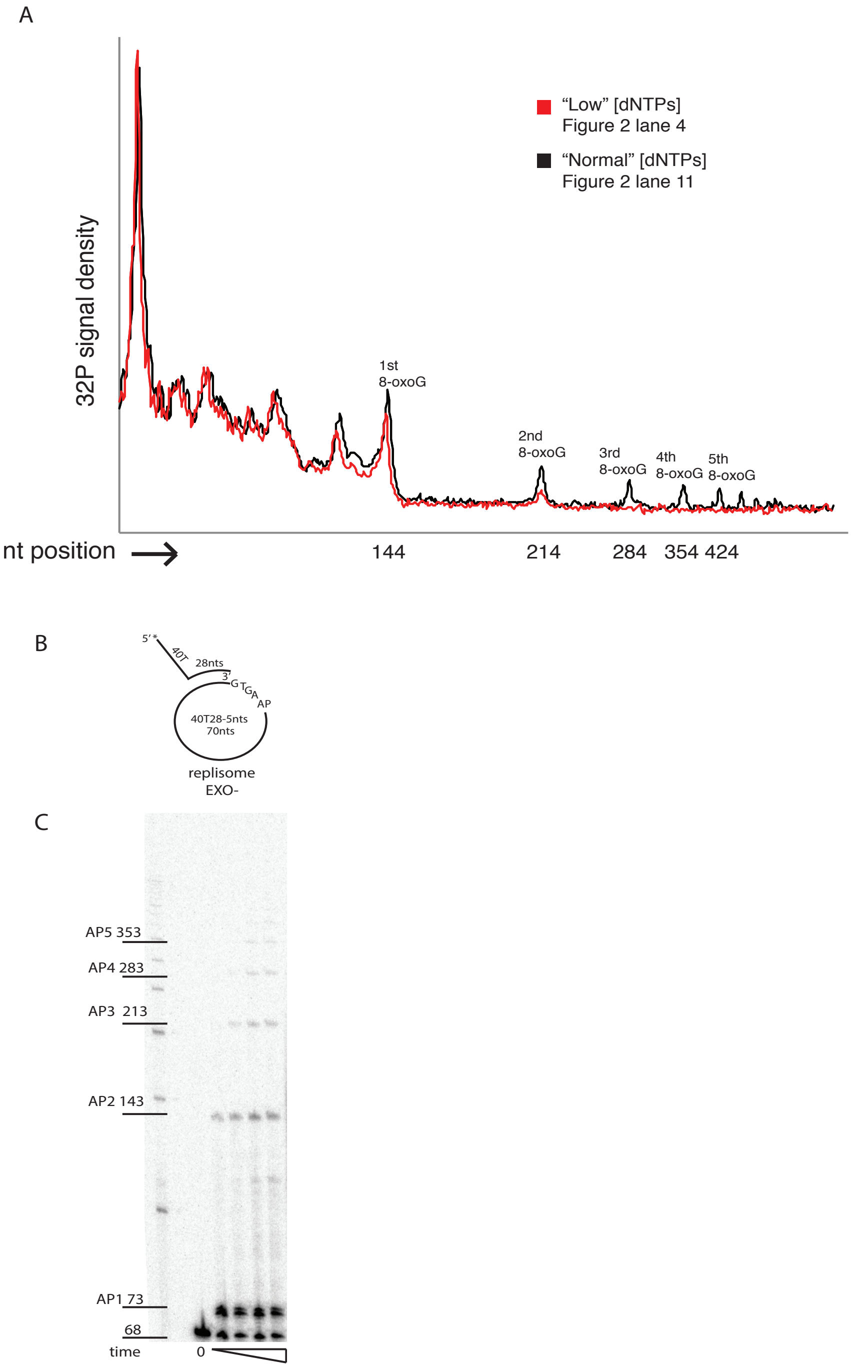

Supplemental Figure 2. Accompanies Figure 2. A) At "normal" dNTP concentrations the mtDNA replication fork is able to produce longer DNA fragments on an 8-oxoG template compared to reaction at "low" dNTP concentrations. A distribution plot of the ^{32}P signal density from Figure 2, lane 4 ("low" dNTPs; red) and Figure 2 lane 11 ("normal" dNTPs; black). The first 5 encounters of 8-oxoG are indicated. The distribution of the ^{32}P signal was analysed for each lane using Image J software. B) Abasic site (AP) DNA substrate used in section C. DNA substrate with 68 nts primer (5' ^{32}P (*) radiolabeled) annealed to a 70-mer AP minicircle template. This generated a DNA substrate that has a 40 nts 5' overhang and a 28 nts dsDNA region with an AP site at the +5 position (from the primer 3'end). C) The exonuclease activity of Pol γA ensures the mtDNA replisome cannot synthesize past an abasic site. DNA replication was done on the minicircle template shown in B. Six percent dPAGE gel of replication time course (0, 10, 30, 60, 90 min) shows that the proofreading deficient mtDNA replisome is capable of limited bypass of abasic sites. The first 5 encounters of the abasic site are indicated (AP1-AP5) and marked with the length of the DNA product.

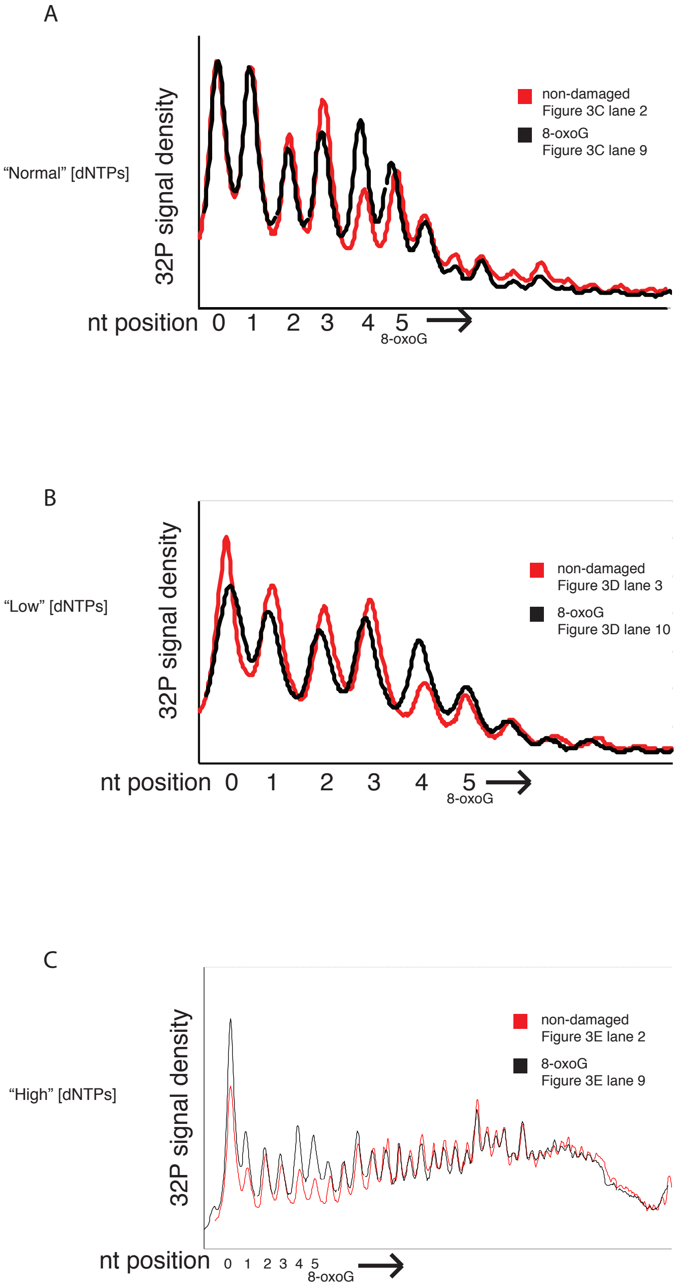

Supplemental Figure 3. Accompanies Figure 3. PrimPol shows modest stalling of DNA synthesis at nt +4, one nt before the 8-oxoG lesion, regardless of dNTP concentrations. Distribution plots of ^{32}P signal density over the indicated lanes in primer extension experiments with PrimPol. A) Figure 3C lane 2 (non-damaged template; red) and lane 9 (8-oxo-G template; black). B) Figure 3D lane 3 (non-damaged template; red) and lane 10 (8-oxo-G template; black). C) Figure 3E lane 2 (non-damaged template; red) and lane 9 (8-oxo-G template; black). The distribution of the ^{32}P signal over each lane was analysed using Image J software.

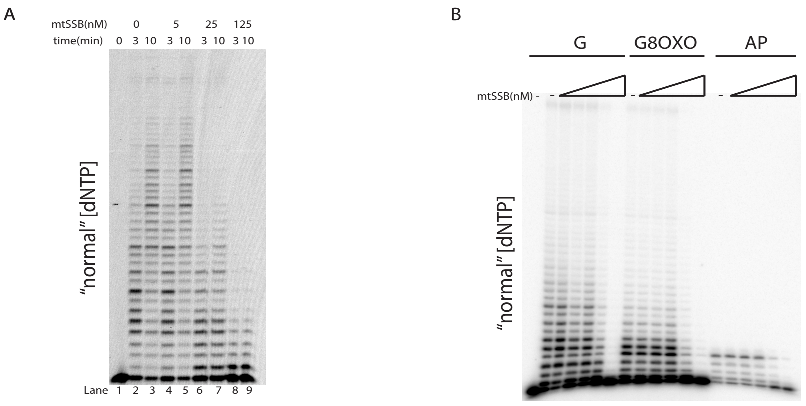

Supplemental Figure 4. MtSSB inhibits PrimPol DNA synthesis on undamaged and oxidatively damaged DNA. A) High concentrations of mtSSB block DNA synthesis by PrimPol. Eleven percent dPAGE separation of a time course replication reaction with 200 nM of PrimPol in the presence "normal" dNTP levels and the indicated amount of tetramer mtSSB. B) dPAGE gel of PrimPol (200 nM) DNA synthesis reaction performed as described in the material and methods with 0, 5, 10 20 125 or 250 nM mtSSB in the presence of "normal" dNTP concentrations on an undamaged, 8-oxo-G or abasic site containing DNA template (as shown in Figure 1A).

Supplemental Table 1A, accompanies Figure 1B. Quantification of signal at damage site (nt positions +3, +4, +5) in reactions with wt Pol γ.

[dNTP]	mtSSB	Template	Signal at +3, +4 and +5 (%)	Lane
Normal	-	G	2	2
Normal	-	G^{8oxo}	8	5
Normal	-	AP	77	8
Normal	+	G	6	10
Normal	+	G^{8oxo}	21	12
Normal	+	AP	73	14
Low	-	G	3	17
Low	-	G^{8oxo}	24	19
Low	-	AP	76	21
Low	+	G	3	23
Low	+	G^{8oxo}	27	25
Low	+	AP	78	27

The ^{32}P signal densities at nt positions +3, +4 and +5 of the 3 minute timepoints from Figure 1B were divided by the signal density of the input primer (lanes 1, 4 and 7 for non-damaged, 8-oxo-G and abasic site templates, respectively). Reactions were performed with the wild-type Pol γ (AB_2).

Supplemental Table 1B, accompanies Figure 1C. Quantification of signal at damage site (nt positions +3, +4, +5) in reactions with exonuclease-deficient Pol γ.

[dNTP]	mtSSB	Template	Signal at +3, +4 and +5 (%)	Lane
Normal	-	G	2	2
Normal	-	G^{8oxo}	5	5
Normal	-	AP	63	8
Normal	+	G	2	10
Normal	+	G^{8oxo}	9	12
Normal	+	AP	104	14
Low	-	G	2	17
Low	-	G^{8oxo}	11	19
Low	-	AP	73	21
Low	+	G	2	23
Low	+	G^{8oxo}	15	25
Low	+	AP	94	27

The ^{32}P signal densities at nt positions +3, +4 and +5 of the 3 minute timepoints from Figure 1B were divided by the signal density of the input primer (lanes 1, 4 and 7 for non-damaged, 8-oxo-G and abasic site templates, respectively). Reactions were performed with the exonuclease-deficient Pol γ (AB_2).

Supplemental Table 2A, accompanies Figure 3C. Quantification of full-length product at "normal" dNTP concentrations.

Template	Time (min)	PrimPol	Pol γ $(AB)_2$	Full-length product (%)	Lane
G	3	-	+	55	4
G	10	-	+	70	5
G	3	+	+	45	6
G	10	+	+	81	7
G^{8oxo}	3	-	+	45	11
G^{8oxo}	10	-	+	63	12
G^{8oxo}	3	+	+	39	13
G^{8oxo}	10	+	+	73	14

^{32}P signal densities from nt positions +67 to +70 relative to the signal of the input primer in a control reaction without added protein (lanes 1 and 8 for non-damaged and 8-oxo-G templates, respectively).

Supplemental Table 2B, accompanies Figure 3D. Quantification of full-length product at low dNTP concentrations.

Template	Time (min)	PrimPol	Pol γ $(AB)_2$	Full-length product (%)	Lane
G	3	-	+	39	4
G	10	-	+	37	5
G	3	+	+	39	6
G	10	+	+	64	7
G^{8oxo}	3	-	+	22	11
G^{8oxo}	10	-	+	19	12
G^{8oxo}	3	+	+	25	13
G^{8oxo}	10	+	+	45	14

^{32}P signal densities from nt positions +67 to +70 relative to the signal of the input primer in a control reaction without added protein (lanes 1 and 8 for non-damaged and 8-oxo-G templates, respectively).

Supplemental Table 2C, accompanies Figure 3E. Quantification of full-length product at high dNTP concentrations.

Template	Time (min)	PrimPol	Pol γ $(AB)_2$	Full-length product (%)	Lane
G	3	-	+	65	4
G	10	-	+	74	5
G	3	+	+	56	6
G	10	+	+	84	7
G^{8oxo}	3	-	+	60	11
G^{8oxo}	10	-	+	70	12
G^{8oxo}	3	+	+	43	13
G^{8oxo}	10	+	+	80	14

^{32}P signal densities from nt positions +67 to +70 relative to the signal of the input primer in a control reaction without added protein (lanes 1 and 8 for non-damaged and 8-oxo-G templates, respectively).

Supplemental Table 2D, accompanies Figure 3E. Quantification of signal at 8-oxo-G site at high dNTP concentrations.

Template	PrimPol	Pol γ $(AB)_2$	+3, +4 and +5 (%)	Lane
G	-	+	1.2	4
G	+	+	2.7	6
G^{8oxo}	-	+	3.5	11
G^{8oxo}	+	+	3.7	13

^{32}P signal densities from nt positions +3, +4 and+5 relative to the signal of the input primer in a control reaction without added protein (lanes 1 and 8 for non-damaged and 8-oxo-G templates, respectively).

Supplemental Table 3, accompanies Figure 5. The percentage of bypass products relative to all products, per lane.

dNTPs	Template	PrimPol	% bypass products	Figure, lane #
Low	G	-	22	5B, 1
Low	G	+	13	5B, 3
Low	G^{8oxo}	-	2	5B, 4
Low	G^{8oxo}	+	1	5B, 6
Low	AP	-	0	5B, 7
Low	AP	+	0	5B, 9
"Normal"	G	-	19	5C, 1
"Normal"	G	+	15	5C, 3
"Normal"	G^{8oxo}	-	7	5C, 4
"Normal"	G^{8oxo}	+	2	5C, 6
"Normal"	AP	-	0	5C, 7
"Normal"	AP	+	0	5C, 9
High	G	-	13	5D, 1
High	G	+	20	5D, 3
High	G^{8oxo}	-	13	5D, 4
High	G^{8oxo}	+	12	5D, 6
High	AP	-	1	5D, 7
High	AP	+	2	5D, 9

The signal of products that had been extended past the first encounter of the damage site (at 144 nt) was divided by the total signal of each lane. Where PrimPol was added, it was present at 200 nM. G, non-damaged; G^{8oxo}, 8-oxo-G; AP, abasic site.

Supplemental Table 4. Primers and DNA template sequences use in this study.

Name	Description	Sequence (5'-3')
70 ND	non-damaged template	GAGGGGTATGTGATGGGAGGGCTA<br>GGATATGAGGTGAGTTGAGTGGAGT<br>TGGAAGTAGGCATACCCCTAT
70 ^{8oxo}G	^{8oxo}G template	GAGGGGTATGTGATGGGAGGGCTA<br>GGATATGAGGTGAGTT^{8oxo}GAGTGGA<br>GTTGGAAGTAGGCATACCCCTAT
70 AP	abasic site template	GAGGGGTATGTGATGGGAGGGCTA<br>GGATATGAGGTGAGTTAPAGTGGAG<br>TTGGAAGTAGGCATACCCCTAT
28-mer	for template circularization	AGGCATACCCCTATGAGTTGGAAGT<br>AGG
25-mer	extension assay primer	ATAGGGGTATGCCTACTTCCAACTC
68-mer	extension assay primer	T40CTCATAGGGGTATGCCTACTTCC<br>AACTC
82-mer	extension assay primer	T40CTCATAGGGGTATGCCTACTTCC<br>AACTCCACTCAACTCACC
BIOHindIII	Biotinylated primer with internal HindIII site	GTAGAAGCTTGATCTACGAGAGTAC<br>TATTAGCTATAGGGGTATGCCTACT<br>TCCAACTC
For70ntseq	PCR forward primer	GTAGAAGCTTGATCTACGAGAG
Rev70ntseq	PCR reverse primer	GAGGGGTATGTGATGGGAGGGCTA<br>GG
M13 rev (-49)	Sequencing primer for pUC19	GAGCGGATAACAATTTCACACAGG
